# Prediction of clinically relevant hyperkalemia in patients treated with peptide receptor radionuclide therapy

**DOI:** 10.1186/s13550-014-0074-y

**Published:** 2014-12-24

**Authors:** Constantin Lapa, Rudolf A Werner, Christina Bluemel, Katharina Lueckerath, Dirk O Muegge, Alexander Strate, Heribert Haenscheid, Andreas Schirbel, Martin S Allen-Auerbach, Ralph A Bundschuh, Andreas K Buck, Ken Herrmann

**Affiliations:** 1 Department of Nuclear Medicine, University Hospital Würzburg, Oberdürrbacher Str. 6, Würzburg 97080, Germany; 2 Institute of Psychology, University of Innsbruck, Innsbruck, Austria; 3 Institute of Clinical Chemistry, University Hospital Würzburg, Oberdürrbacher Str. 6, Würzburg 97080, Germany; 4 Ahmanson Translational Imaging Division, Department of Molecular and Medical Pharmacology, David Geffen School of Medicine at UCLA, 10833 Le Conte Avenue, Los Angeles 90095, CA, USA; 5 Department of Nuclear Medicine, University Hospital Bonn, Sigmund-Freud-Straße 25, Bonn 53127, Germany

**Keywords:** NET, PRRT, Hyperkalemia, Kidney function, MAG3, Amino acids

## Abstract

**Background:**

Peptide receptor radionuclide therapy (PRRT) is applied in patients with advanced neuroendocrine tumors. Co-infused amino acids (AA) should prevent nephrotoxicity. The aims of this study were to correlate the incidence of AA-induced hyperkalemia (HK) (≥5.0 mmol/l) and to identify predictors of AA-induced severe HK (>6.0).

**Methods:**

In 38 patients, standard activity of ^177^Lu-labelled somatostatin analogs was administered. Pre-therapeutic kidney function was assessed by renal scintigraphy and laboratory tests. For kidney protection, AA was co-infused. Biochemical parameters (potassium, glomerular filtration rate, creatinine, blood urea nitrogen (BUN), sodium, phosphate, chloride, and lactate dehydrogenase (LDH)) were obtained prior to 4 and 24 h after the AA infusion. Incidence of HK (≥5.0) was correlated with pre-therapeutic kidney function and serum parameters. Formulas for the prediction of severe hyperkalemia (>6.0) were computed and prospectively validated.

**Results:**

At 4 h, HK (≥5.0) was present in 94.7% with severe HK (>6.0) in 36.1%. Values normalized after 24 h in 84.2%. Pre-therapeutic kidney function did not correlate with the incidence of severe HK.

Increases in K^+^ were significantly correlated with decreases in phosphate (*r* = −0.444, *p* < 0.005) and increases in BUN (*r* = 0.313, *p* = 0.056). A baseline BUN of >28 mg/dl had a sensitivity of 84.6% and a specificity of 60.0% (AUC = 0.75) in predicting severe HK of >6.0 (phosphate, AUC = 0.37).

Computing of five standard serum parameters (potassium, BUN, sodium, phosphate, LDH) resulted in a sensitivity of 88.9% and a specificity of 79.3% for the prediction of severe HK >6.0 (accuracy = 81.6%).

**Conclusions:**

A combination of serum parameters predicted prospectively the occurrence of relevant HK with an accuracy of 81.6% underlining its potential utility for identifying ‘high-risk’ patients prone to PRRT.

## Background

Peptide receptor radionuclide therapy (PRRT) with radiolabelled somatostatin agonists such as ^90^Y- or ^177^Lu-labelled DOTATOC/-TATE ([1,4,7,10-tetraazacyclododecane-NN′,N″,N′″-tetraacetic acid]-d-Phe1,Tyr3-octreotoc/-tate) is routinely used for advanced and/or metastatic neuroendocrine tumors (NET) which overexpress somatostatin receptor subtype II (SSTR II) [1]-[3]. Although the treatment is generally well tolerated, side effects like nausea, myelosuppression, and renal failure have been reported [4],[5]. Tubular reabsorption and retention of the radiopeptide at the proximal tubule may lead to an excessive radiation dose to the kidney. Therefore, pre-therapeutic assessment of renal function is mandatory and is routinely assessed by means of laboratory tests (creatinine, glomerular filtration rate (GFR), and blood urea nitrogen (BUN)) or nuclear imaging techniques (e.g., renal scintigraphy with ^99m^Tc-mercapto-acetylglycyl-glycyl-glycine [^99m^Tc-MAG3]).

For kidney protection, various protocols using co-infusion of positively charged amino acids (AA) like l-arginine and l-lysine have been introduced [6]-[10]. However, side effects including nausea, vomiting, and clinically relevant hyperkalemia have been described [11],[12]. In a recent study including patients with NET undergoing PRRT with ^90^Y-DOTATOC, Giovacchini et al. reported the incidence of hyperkalemia (>5.0 mmol/l) in more than three-fourths of patients with potassium levels up to 6.7 mmol/l, thereby raising awareness of this potentially life-threatening adverse effect induced by AA-coinfusion [13].

In clinical routine practice, early or even pre-therapeutic identification of patients at risk for developing severe hyperkalemia (>6.0 mmol/l) due to AA-coinfusion would be of high relevance. Therefore, the aims of this study were to assess the incidence and severity of hyperkalemia (≥5.0 mmol/l) and to identify predictors of PRRT-related severe hyperkalemia (>6.0 mmol/l).

## Methods

### Study design

The study consists of a retrospective and a prospective part. In the retrospective analysis, 38 consecutive patients were included. Incidence and severity of hyperkalemia (≥5.0 mmol/l) were correlated with pre-therapeutic kidney function. Three formulas for the prediction of severe hyperkalemia (>6.0 mmol/l) were computed retrospectively from different combinations of serum parameters. Thereafter, these formulas were prospectively validated in a cohort including another 38 patients.

This study comprised of only retrospective analysis of routinely acquired data and therefore the institutional review board (ethics committee of the Medical Faculty of the University of Würzburg) waived the requirement for additional approval. All patients gave written informed consent to receive standard-of-care PRRT. In October 2013, the routinely administered amount of AA was reduced from 75 g to 50 g to comply with the most recent version of the joint International Atomic Energy Agency (IAEA), European Association of Nuclear Medicine (EANM), and Society of Nuclear Medicine and Molecular Imaging (SNMMI) practical guidance [6].

### Patients

From July 2013 to December 2013, 38 consecutive patients (24 males, 14 females) referred for PRRT were enrolled. Their mean age was 62 ± 14 years (range, 24 to 82). The general exclusion criteria, as defined by the joint IAEA, EANM, and SNMMI practical guidance, were applied [6]. All patients had progressive disease. Out of the 38 subjects enrolled, 7 received their first treatment cycle. The following risk factors for the occurrence of kidney toxicity were assessed in all patients: diabetes mellitus (*n* = 5) and arterial hypertension (*n* = 12). Additionally, potassium-sparing anti-hypertensive medications and long-term use of NSAIDs (*n* = 17) were recorded. Pretherapeutic ECG was unremarkable in all patients. None of the subjects had a history of arrhythmogenic diseases.

From January 2014 to April 2014, the formulas for hyperkalemia-risk-assessment derived from the retrospective cohort were validated prospectively in 38 additional consecutive subjects (23 males, 15 females; mean age 58 ± 12 years (range, 24 to 76)). Twenty-two patients were included and monitored during both treatment cycles with co-infusion of 75 g and 50 g of AA, as previously reported [14]. Detailed patient characteristics are given in Table 1.

**Table 1:** Patient characteristics

	**Retrospective cohort**	**Prospective cohort**
Number of subjects	38	38
Mean age (years)	62 ± 14	58 ± 12
Sex	24 males, 14 females	23 males, 15 females
Number of treatment cycle, mean	3.26 ± 2.00	4.26 ± 2.44
Mean activity (GBq)	23.91 ± 14.67 ^177^Lu-DOTATATE	31.68 ± 19.21 ^177^Lu-DOTATOC
Primary tumor		
Pancreatic NET	9	10
Small bowel NET	5	4
Ileum NET	4	6
Cerebral tumor	4	2
NET of unknown origin	4	2
Gastric NET	2	1
MTC	2	2
Caecum NET	2	2
Rectal NET	1	2
Liver NET	1	2
Thymus NET	1	1
Paraganglioma	1	1
Lung NET	1	3
Hemangioendothelioma	1	

Main baseline features of the patients enrolled in the study.

### Assessment of kidney function

About 2 weeks prior to PRRT, 22 patients underwent renal scintigraphy with ^99m^Tc-labelled mercaptoacetyltriglycine (MAG3) on a single head gamma camera (Signature; Siemens, Erlangen, Germany) equipped with low-energy, high-resolution collimators. The radiotracer was prepared using a commercially available kit (MAG3, Mallinckrodt Pharmaceuticals, Neustadt an der Donau, Germany). All patients were asked to drink at least 250 ml of water 30 min prior to the examination. Imaging started immediately after injection of 98 ± 6 MBq ^99m^Tc-MAG-3 and was performed according to national guidelines [15]. Venous blood samples were drawn from the arm contralateral to the injection site 20 and 30 min after tracer injection to allow for calculation of tubular extraction rates (TER). Sixteen patients underwent renal assessment by ^131^I-hippurate on an outpatient basis and were excluded from this part of the study.

### Therapy

#### Preparation of ^177^Lu-DOTATATE/-TOC and PRRT

^177^Lu-DOTATATE/-TOC was prepared with minor modifications as previously reported [14],[16]. All patients were admitted 1 day prior to therapy to guarantee adequate hydration (1-l saline) and were hospitalized for a total of 3 days. In the retrospective group, 37.5 g l-arginine hydrochloride and 37.5 g l-lysine hydrochloride (75 g total amount of AA, pH 7.0) were intravenously administered over 4 h beginning 30 min to 1 h prior to PRRT. In the prospective cohort, amounts of AA infused were changed to 25 g of each l-arginine and l-lysine (pH 7.0; single-day 50 g protection-protocol) in order to comply with the joint IAEA, EANM, and SNMMI practical guidance [6]. No other fluids were administered. As recently published, the two different amounts of AA do not result in any difference in incidence or severity of hyperkalemia [14].

#### Pre- and post-therapeutic blood samples

One day before, 4 and 24 h after the beginning of the AA infusion, standard blood values (potassium, glomerular filtration rate, creatinine, BUN, sodium, phosphate, chloride [available in all patients] and lactate dehydrogenase [retrospective cohort: available in *n* = 36/38, prospective cohort: all patients]) were assessed. Application of the tourniquet was used as short as possible to minimize false-positive potassium values due to hemolysis. Serum potassium levels were measured with the indirect ion sensitive electrode (ISE) Cobas 8000 system (Roche Diagnostics, Mannheim, Germany). All samples were screened for hemolysis. Absorbance of the diluted serum samples (dilution 1:26) was measured at 570 (primary wave length) and at 600 nm (secondary wave length), and hemolysis indices were calculated according to the manufacturer’s instruction. Serum samples with hemolysis indices above 90 (equaling 90 mg/dl free hemoglobin) were considered to be hemolytic and excluded from further evaluation (<3% of all samples drawn).

#### Analysis and statistics

Student’s *t* test was used to test for simple differences between patients with or without hyperkalemia in various blood parameters. A *p* value of 0.05 or less was considered to be significant. Moreover, we have assumed that some blood parameters promote and some prevent hyperkalemia. Therefore, it may be possible that the human body tries to restore a homeostatic imbalance by increasing one blood parameter or decreasing another. Such relationships could not be accounted for by Student’s *t* tests. However, linear discriminant analysis makes it possible to classify patients with different conditions (e.g., hyperkalemia) on the basis of a set of predictors (e.g., multiple blood parameters). Hence, we performed multiple linear discriminant analysis to improve the prediction of severe hyperkalemia (>6.0 mmol/l) by calculating linear combinations of the relevant blood parameters. In a backward stepwise analysis, the weakest serum predictor contributing least to the prediction of severe hyperkalemia was excluded according to its *p* value. Cutoff values for the prediction of severe hyperkalemia were determined for each formula by receiver operating characteristic (ROC) analysis [17].

Additionally, in order to test a correlation between increases of potassium and clinical factors, Pearson’s correlation was performed. A *p* value of 0.05 or less was considered to be significant.

## Results

### AA-induced hyperkalemia (retrospective cohort)

At baseline, mean K^+^ levels were 4.41 ± 0.37 mmol/l. 4 h after PRRT, mean serum potassium level increased to 5.90 ± 0.80 mmol/l with values ≥5.0 mmol/l in 36/38 subjects (94.7%; 17/36 subjects with history of potassium-sparing anti-hypertensive medications and long-term use of NSAIDs). Thirteen of those 36 patients (36.1%) experienced severe hyperkalemia >6.0 mmol/l. Only 2/38 subjects did not show elevated K^+^ levels.

Five of the 13 subjects (38.5%, 2/5 with diabetes) with severe hyperkalemia became symptomatic with palpitations and general malaise. In all five patients, ECG revealed flattened *P* waves and high peaked *T* waves with peak serum potassium levels of 8.5 mmol/l. These patients required immediate therapy to lower serum potassium concentrations. We effectively administered 10 units of regular insulin in 500 ml of 10%dextrose over 60 min.

**Figure 1:**
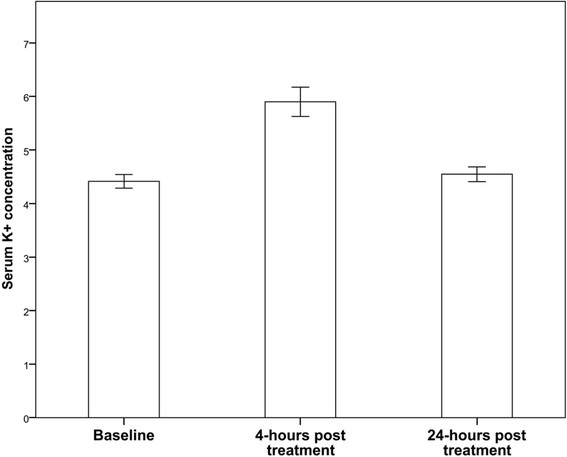
**Serum potassium concentrations (mmol/l) at baseline, 4 and 24 h after PRRT (retrospective cohort,*n*** **= 38).**

At 24 h, K^+^ levels had almost returned to baseline levels (mean 4.55 ± 0.42 mmol/l, *p* > 0.05 compared to baseline; *p* < 0.001 compared to 4 h) in 32/38 (84.2%) subjects. Six of the 38 patients (15.8%) still demonstrated slightly elevated potassium levels with a maximum of 5.5 mmol/l (Figure 1; Table 2).

**Table 2:** Biochemical parameters of patients at baseline, 4 and 24 h after PRRT

	**Normal range**	**Baseline**	**4 h**	**24 h**
Potassium (mmol/l)	3.5 to 5.0	4.41 ± 0.37	5.9 ± 0.8	4.55 ± 0.42
GFR (CKD-EPI) (ml/min/1.73 m^2^)	-	82.79 ± 25.49	80 ± 23.81	81.22 ± 26.34
Creatinine (mg/dl)	0 to 0.95	0.93 ± 0.28	0.95 ± 0.26	0.97 ± 0.29
BUN (mg/dl)	10 to 50	33.32 ± 12.89	42.38 ± 12.22	36.95 ± 14.07
Sodium (mmol/l)	135 to 145	138.79 ± 3.03	133.63 ± 3.11	137.42 ± 14.07
Phosphate (mmol/l)	0.87 to 1.45	1.07 ± 0.19	0.84 ± 0.16	1.21 ± 0.22
Chloride (mmol/l)	94 to 110	99.75 ± 4.3	107.84 ± 3.7	105.32 ± 4.15
LDH (U/l)	≤250	218.87 ± 59.98	220.38 ± 55.2	219.36 ± 45.21

Biochemical parameters of patients at baseline, 4 and 24 h after PRRT (retrospective cohort; LDH, *n* = 36/38; all other parameters, *n* = 38). Values are given as mean ± SD.

### Correlation of hyperkalemia to kidney function and clinical factors (retrospective cohort)

All patients presented with normal kidney function according to ^99m^Tc-MAG3 scintigraphy with a mean TER of 231 ± 47 ml/min/1.73 m^2^. The pre-therapeutic TER did not correlate with the post-therapeutic potassium level (*r* = −0.120) and was unable to predict incidence or severity of hyperkalemia. No cutoff value of minimum glomerular filtration rate excluding severe hyperkalemia could be derived from serum blood samples (*r* = −0.055, *p* = 0.74).

Additional serum parameters (potassium, BUN, sodium, phosphate, chloride, LDH) were analyzed (Table 2). Out of all variables investigated, a pre-therapeutic BUN cutoff value of >28 mg/dl (defined by ROC analysis) had a sensitivity of 84.6% and a specificity of 60% in predicting severe hyperkalemia ≥6.0 mmol/l (area under the curve, AUC = 0.75). No clinical characteristic including patient age, cumulative dose, number of treatment cycles, potentially interfering medications, or clinical risk factors correlated with the incidence or severity of post-treatment hyperkalemia (Additional file 1: Table S1).

**Figure 2:**
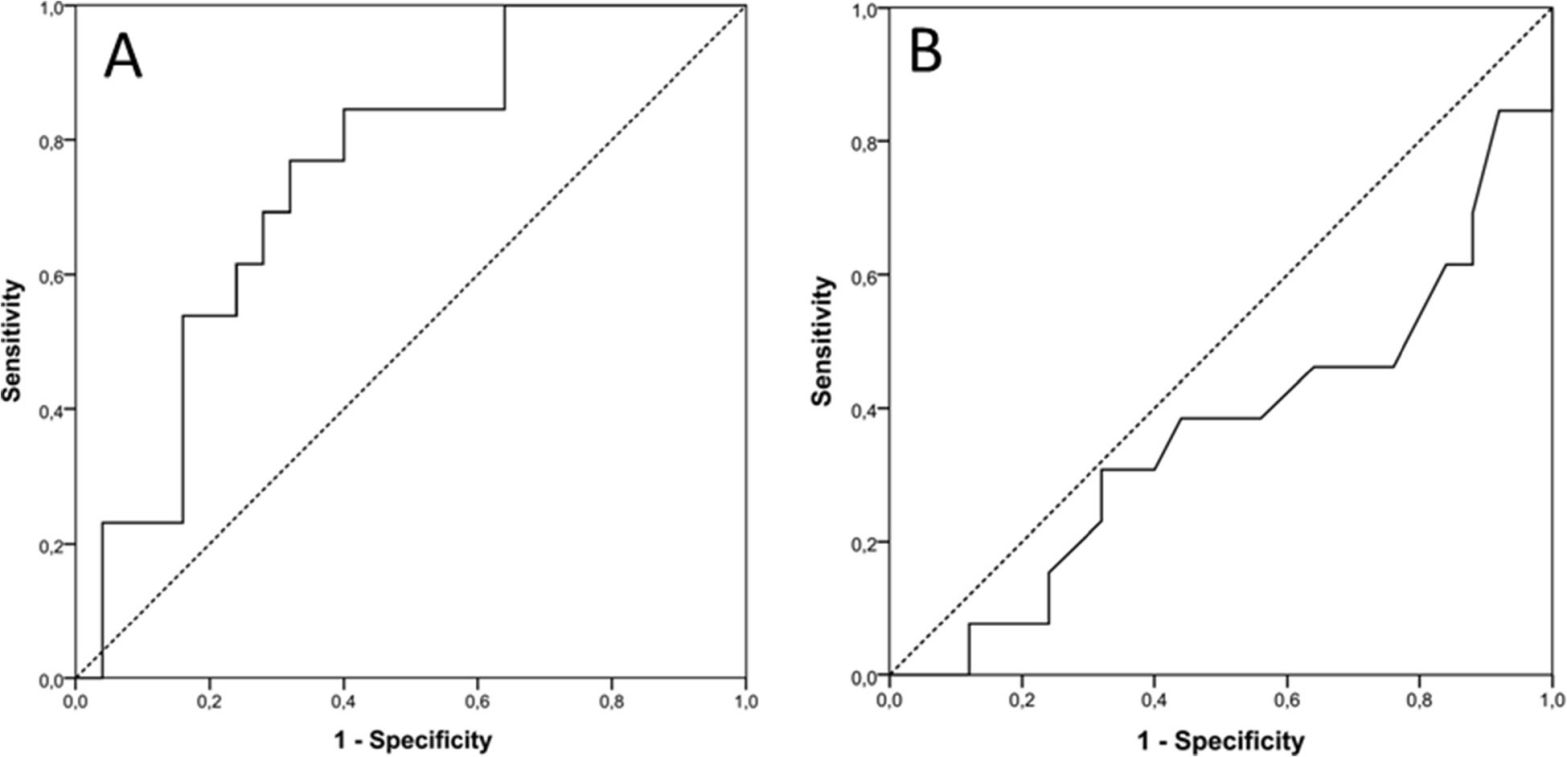
**Receiver operating characteristic curves for blood urea nitrogen and phosphate.** Receiver operating characteristic (ROC) curves for **(A)** serum blood urea nitrogen (BUN) and **(B)** phosphate for the prediction of severe hyperkalemia (>6.0 mmol/l) after peptide receptor radionuclide therapy (retrospective cohort, *n* = 38). BUN demonstrates some value as a single, simply achievable parameter (area under the curve [AUC] = 0.75) whereas serum phosphate is not a suitable predictor (AUC = 0.37).

We also investigated changes to the baseline to 4-h post-therapy initiation. Changes in serum BUN tended to positively correlate with hyperkalemia (*r* = 0.313; *p* < 0.056) (Figure 2A). A significant negative correlation between increase in serum potassium and phosphate was observed (*r* = −0.444; *p* < 0.005). However, this parameter did not predict severe hyperkalemia (>6.0 mmol/l) (AUC = 0.37) (Figure 2B).

### Calculation of formulas for hyperkalemia-risk-assessment (retrospective cohort)

In order to improve estimation of the risk of severe hyperkalemia, several formulas were generated based on an arbitrarily set cutoff potassium level >6.0 mmol/l and different serum parameters. Three of these formulas showed superior results in retrospective evaluation.

The parameters used in the formulas are the pre-therapeutic plasma concentrations of potassium (K^+^, in mmol/l, available in 38/38 patients), sodium (Na^+^, in mmol/l, *n* = 38/38), phosphate (PO4^2−^, in mmol/l, *n* = 38/38), creatinine (Crea, in mg/dl, *n* = 38/38), blood urea nitrogen (BUN, in mg/dl, *n* = 38/38), lactate dehydrogenase (LDH, in U/l, *n* = 36/38), as well as the glomerular filtration rate (GFR, in ml/min/1.73 m^2^, *n* = 38/38).

The first formula included all seven parameters:

Formula 1 (*n* = 36):

$$X=1.6\cdot K^{+}+0.055\cdot \mathrm{BUN}+0.004\cdot LDH-\left(0.243\cdot {Na}^{+}+3.922\cdot PO4^{2-}+0.009\cdot GFR+1.358\cdot \mathrm{Crea}\right)$$

Defining the cutoff as *X* ≥ −29.909 resulted in a sensitivity of 84.6% and a specificity of 82.6%, respectively (AUC = 0.92, accuracy = 83.3%).

GFR and creatinine were omitted in Formula 2:

Formula 2 (*n* = 36):

$$X=1.742\cdot K^{+}+0.045\cdot \mathrm{BUN}+0.003\cdot LDH-\left(0.252\cdot {Na}^{+}+\;3.927\cdot PO4^{2-}\right)$$

Based on an AUC of 0.90 for Formula 2, the optimal cutoff value was defined as *X* ≥ −29.226 with a sensitivity of 92.3% and a specificity of 82.6% (accuracy = 86.1%).

Excluding further two parameters (BUN, LDH) while introducing creatinine led to Formula 3:

Formula 3 (*n* = 38):

$$X=2.046\cdot K^{+}+1.053\cdot \mathrm{Crea}-\left(0.305\cdot {Na}^{+}+4.356\cdot PO4^{2-}\right)$$

**Figure 3:**
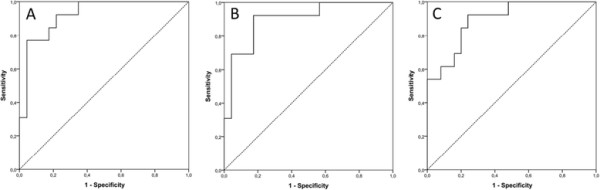
**Receiver operating characteristic curves for the prediction of severe hyperkalemia (>6.0 mmol/l).** Receiver operating characteristic (ROC) curves for the three formulas computed for the prediction of severe hyperkalemia (>6.0 mmol/l) with **(A)** seven-, **(B)** five-, and **(C)** four-serum parameters. Pre-therapeutic plasma concentrations of potassium, sodium, phosphate, creatinine, blood urea nitrogen, lactate dehydrogenase, as well as the glomerular filtration rate were included. All formulas show excellent areas under the curve ranging from 0.90 to 0.92.

ROC analysis revealed an AUC of 0.90. A cutoff value of *X* ≥ −37.035 resulted in a sensitivity of 92.3% and a specificity of 72.0% in excluding severe hyperkalemia (accuracy = 78.9%). The respective ROC curves are given in Figure 3.

### Validation of formulas for hyperkalemia-risk-assessment (prospective cohort)

In a prospective setup, additional 38 patients were enrolled to validate the formulas: none of the patients suffered from hyperkalemia prior to PRRT with mean potassium levels of 4.34 ± 0.39 mmol/l. 4 h after PRRT, mean K^+^ level was 5.69 ± 0.61 mmol/l with values ≥5.0 mmol/l in 28/38 subjects (73.6%; 15/28 subjects with history of potassium-sparing anti-hypertensive medications and long-term use of NSAIDs). Nine of the 38 patients (23.7%) experienced severe hyperkalemia >6.0 mmol/l, and three patients became symptomatic with palpitations and ECG changes. After 24 h, K^+^ levels returned to baseline levels (mean 4.53 ± 0.37 mmol/l).

The three formulas were validated using the cutoff values mentioned above. Formula 1 showed a sensitivity of 77.8% and a specificity of 79.3% (accuracy = 78.9%) and Formula 2 had a sensitivity of 88.9% and a specificity of 79.3% (accuracy = 81.6%). Formula 3, including four blood values, could predict severe hyperkalemia >6.0 mmol/l with a sensitivity of 66.7% and a specificity of 82.8% (accuracy = 78.9%).

An overview over the results of the three formulas is given in Table 3 (retrospective and prospective cohort).

**Table 3:** Overview of results (retrospective and prospective cohort)

	**Retrospective cohort (75 g AA)**			**Prospective cohort (50 g AA)**		
**Formula**	**Sensitivity**	**Specificity**	**Accuracy**	**Sensitivity**	**Specificity**	**Accuracy**
1	84.6 (11/13)	82.6 (19/23)	83.3 (30/36)	77.8 (7/9)	79.3 (23/29)	78.9 (30/38)
2	92.3 (12/13)	82.6 (19/23)	86.1 (31/36)	88.9 (8/9)	79.3 (23/29)	81.6 (31/38)
3	92.3 (12/13)	72.0 (18/25)	78.9 (30/38)	66.7 (6/9)	82.8 (24/29)	78.9 (30/38)

Overview of results (retrospective cohort, Formulas 1 and 2: *n* = 36; Formula 3: *n* = 38; left; prospective cohort, all formulas: *n* = 38, right). Sensitivity, specificity, and accuracy are given in percentage. Numbers in brackets indicate patient numbers. AA = amino acid solution.

## Discussion

The high incidence of PRRT-related hyperkalemia in patients treated for NET has been recently reported [13]. The aim of this study was to identify pre-therapeutic parameters for prediction of AA-induced hyperkalemia in patients undergoing PRRT. Combinations of serum parameters were retrospectively identified, and their predictive value was prospectively evaluated. No single serum parameter could reliably predict the occurrence of hyperkalemia. In the prospective validation cohort, a formula including five routine biochemical serum parameters (potassium, BUN, sodium, phosphate, LDH; Formula 2) correctly identified 8 of 9 patients with (sensitivity: 88.9%) and 23 of 29 patients without hyperkalemia >6.0 mmol/l (specificity: 79.3%); resulting in an accuracy of 31 of 38 (81.6%).

Showing the highest sensitivity and a false-negative rate of 11.1%, thus really identifying patients suffering from clinically relevant hyperkalemia, Formula 2 seems to be the most promising. Considering a potentially life-threatening side-effect, this approach might be a quite valuable tool for the clinical physician planning PRRT.

Arginine monohydrochloride and lysine monohydrochloride, both cationic amino acids, are known to cause transient hyperkalemia by driving potassium from the intracellular to the extracellular compartment [18],[19]. In our cohort, hyperkalemia (>5.0 mmol/l) was induced in almost every patient and K^+^ levels rose by 33.8%. This finding is in line with previous results [11],[13],[20]. Thus, close monitoring of patients after PRRT and evaluation of serum potassium levels should be mandatory as more than 35% of subjects (13/36) experienced rises in K^+^ to > 6.0 mmol/l. Five patients became symptomatic and required potassium-lowering interventions (e.g. insulin with glucose). Of note, two of those patients suffered from diabetes (40% of all diabetic patients in this cohort), a sub-cohort maybe especially prone to post-therapeutic hyperkalemia due to reduced sensitivity to endogenous insulin. An earlier study has already reported on this pathophysiologic mechanism [21]. In our study, no significant correlation between diabetes and incidence or severity of increases in potassium could be detected; however, this might be related to small sample sizes.

To prevent potential complications, we aimed to identify predictors for severe hyperkalemia >6.0 mmol/l to help the clinician in identifying patients at risk. The only factor with limited predictive value was an increased serum BUN. A cutoff value of >28 mg/dl provided a sensitivity of 84.6% but a rather low specificity of 60.0% in excluding severe hyperkalemia >6.0 mmol/l (AUC = 0.75). However, since emphasis should be put on high sensitivity, this marker is suggested as simple first test in evaluating potentially severe hyperkalemia.

In a more sophisticated approach, a combination of biochemical markers which are all affected by PRRT was used to derive three different formulas for predicting the risk of AA-induced hyperkalemia. The four-to-seven parameters considered by these formulas can be obtained from one blood sample. All formulas showed excellent areas under the curve in retrospective ROC analysis (range, 0.89 to 0.92).

In order to allow broad applicability, accuracy was prospectively investigated. In this cohort, the formula taking into account five serum parameters (potassium, creatinine, sodium, LDH and phosphate; Formula 2) demonstrated again both excellent sensitivity (88.9%) and good specificity (79.3%).

The following limitations have to be considered: in order to strengthen the statistical power and the prognostic significance of our findings, further studies with larger patient cohorts need to be performed. Secondly, almost more than half of the patients were included and monitored during both retrospective and prospective treatment cycles with co-infusion of 75 or 50 g of AA. Moreover, the amount of AA routinely administered was reduced from 75 to 50 g to comply with the latest joint IAEA, EANM, and SNMMI practical guidance in October 2013 [6]. Thus, the retrospectively calculated formulas were derived administering 75 g AA whereas in the prospective part these findings were validated administering 50 g AA. However, in our intra-individual comparison of both protocols, incidence and severity of hyperkalemia do not significantly vary between 75 and 50 g AA, respectively [14]. Due to this finding of our previous study, a broad applicability in clinical routine is independent from AA dosing (75 or 50 g, respectively). On a final note, different radiolabelled compounds were used. In the prospective cohort, DOTATATE was replaced by DOTATOC. Esser and colleagues reported that therapy with ^177^Lu-octreotate is preferable due to its higher tumor residence time. However, a Dutch research group did not report on incidence of hyperkalemia after administering both protocols [22].

In conclusion, the high incidence of hyperkalemia in almost all patients undergoing PRRT requires a high degree of clinical attention. Serum blood sampling after treatment is mandatory, given that more than 20% of patients experience a potentially life-threatening rise in K^+^ values to >6.0 mmol/l. Early identification of subjects at risk who might require immediate therapeutic intervention would be of great value. To the best of our knowledge, this is the first attempt to derive a robust estimate of the risk of AA-induced hyperkalemia in patients scheduled for PRRT. With an accuracy of 81.6% and a low false-negative rate, we established a reliable prospectively validated formula assessed by one simple blood collection selecting ‘high risk’ patients being vulnerable for severe hyperkalemia. In general, this formula might be a valuable tool for the clinical physician planning PRRT and was recently implemented into our clinical routine.

## Conclusions

No single serum-based factor can reliably predict post-therapeutic increases in potassium to values >6.0 mmol/l after PRRT. Our data suggest that prediction of severe hyperkalemia using a combination of routine serum parameters prior to PRRT including potassium, creatinine, sodium, LDH, and phosphate identified ‘high risk’ patients with an accuracy of 81.6%. We therefore conclude that this formula is a valuable tool for identifying patients prone to develop PRRT-associated severe hyperkalemia.

## Competing interests

The authors declare that they have no competing interests.

## Authors’ contributions

CL, RAW, KL, AnS, AKB, MAA, and KH conceived the study. CB and AlS acquired the data. CL, RAW, HH, and DOM analyzed and interpreted the data. CL, RAW, CB, and AlS were involved in the drafting of the manuscript; and KL, AnS, AKB, RAB, CB, and KH revised it critically for important intellectual content. All authors gave final approval of the version to be published and agree to be accountable for all aspects of the work in ensuring that questions related to the accuracy or integrity of any part of the work are appropriately investigated and resolved.

## Additional file

## Supplementary Material

Additional file 1 Clinical risk factors correlated with the incidence or severity of post-treatment hyperkalemia. (DOCX 14 kb) Click here for file
