# Supplementary material for: Prediction of clinically relevant hyperkalemia in patients treated with peptide receptor radionuclide therapy
Source: EJNMMI Res. 2014 Dec 24;4:74. doi: 10.1186/s13550-014-0074-y (PMC4412196; doi:10.1186/s13550-014-0074-y)
Supplement: Additional file 1 — Clinical risk factors correlated with the incidence or severity of post-treatment hyperkalemia. (DOCX 14 kb) [file s13550-014-0074-y-S1.docx]

Table S1

| K^+^ level: baseline-4 h | Number of Tx cycle | Cumulative dose | ^99m^Tc-MAG3 clearance | Age | K^+^-influencing drugs | Clinical risk factors |
| --- | --- | --- | --- | --- | --- | --- |
| r-value  p-value | -0.16  0.35 | -0.13  0.43 | -0.02  0.93 | 0.18  0.28 | 0.22  0.18 | 0.02  0.91 |
